# Biomechanical Voice Parameters as Potential Biomarkers for Phenotype Differentiation in Amyotrophic Lateral Sclerosis: A Cross-Sectional Study

**DOI:** 10.3390/medsci14010112

**Published:** 2026-02-26

**Authors:** Margarita Pérez-Bonilla, Marina Mora-Ortiz, Paola Díaz-Borrego, María Nieves Muñoz-Alcaraz, Fernando J. Mayordomo-Riera, Eloy Girela-López

**Affiliations:** 1 Interlevel Clinical Management Unit of Physical Medicine and Rehabilitation, Reina Sofia University Hospital, Cordoba and Guadalquivir Health District, 14011 Cordoba, Spain; sr1maif@uco.es; 2 Department of the Applied Physics, Radiology and Physical Medicine, Faculty of Medicine and Nursing of Córdoba, University of Córdoba, 14004 Cordoba, Spain; 3 Maimonides Biomedical Research Institute of Córdoba (IMIBIC), Reina Sofía University Hospital, University of Córdoba, 14004 Cordoba, Spain; marina.mora@imibic.org; 4 Physical Medicine & Rehabilitation, Virgen Macarena University Hospital, 41009 Seville, Spain; paola.diaz.sspa@juntadeandalucia.es; 5 Department of Morphological and Sociosanitary Sciences, Faculty of Medicine and Nursing of Córdoba, University of Córdoba, 14004 Cordoba, Spain; ft1gilpe@uco.es

**Keywords:** amyotrophic lateral sclerosis, bulbar-onset ALS, voice analysis, acoustic voice measures, biomechanical voice analysis, phonatory function, potential biomarkers, clinical phenotypes

## Abstract

**Background/Objectives**: Amyotrophic lateral sclerosis (ALS) is a clinically heterogeneous neurodegenerative disease in which bulbar involvement frequently affects speech and voice production. Although acoustic voice analysis can detect phonatory alterations in ALS, its ability to differentiate clinical phenotypes remains limited. This study investigated whether biomechanical voice parameters provide complementary information for characterizing bulbar involvement across bulbar-onset ALS (ALS-B) and spinal-onset ALS (ALS-S) and explored their association with clinical and functional measures. **Methods**: This cross-sectional observational study included 50 patients with ALS (20 ALS-B, 30 ALS-S) and 50 controls with non-neurological voice disorders. Sustained vowel phonation was analyzed using acoustic measures and biomechanical voice parameters derived from a standardized model of vocal fold vibration. Perceptual voice severity was assessed using the GRBAS scale, while functional status was evaluated with the ALS Functional Rating Scale–Revised (ALSFRS-R) and the Barthel Index. Associations with clinical measures were explored in secondary analyses. **Results**: Compared with controls, ALS patients showed significant differences in acoustic measures and several biomechanical parameters related to glottal closure and vibratory stability. Biomechanical analysis revealed significant differences between ALS-B and ALS-S, particularly in parameters reflecting vibratory asymmetry, glottal tension and cycle-to-cycle instability. Unexpectedly, ALS-B showed greater perceptual voice severity and higher Barthel Index scores than ALS-S, while no differences were observed in global ALSFRS-R total scores. **Conclusions**: Biomechanical voice analysis appears to capture physiologically meaningful alterations in vocal fold function in ALS and provides complementary information for characterizing bulbar motor involvement across clinical phenotypes, particularly ALS-B disease. When combined with acoustic and clinical assessments, this approach may enhance the evaluation of bulbar involvement and functional status in ALS.

## 1. Introduction

ALS is a progressive and fatal neurodegenerative disease characterized by the degeneration of upper and lower motor neurons, leading to progressive muscle weakness, functional decline, and ultimately respiratory failure. The disease is clinically heterogeneous, with substantial variability in age at onset, progression rate, and pattern of motor involvement. Among the different clinical phenotypes, ALS-B is consistently associated with earlier functional deterioration and poorer prognosis compared with limb-onset forms [1]. Despite advances in the understanding of ALS pathophysiology, diagnosis remains primarily clinical, and no single objective biomarker is currently available to reliably capture disease onset or progression, particularly in the early stages [2].

Bulbar dysfunction represents a prominent and often early manifestation of ALS, frequently affecting speech and voice production. Dysarthria may be the initial presenting symptom in up to 25–30% of patients and develops in the majority of individuals as the disease progresses, with a substantial impact on communication and quality of life [3]. Importantly, alterations in voice production may precede overt neurological signs or perceptually evident dysarthria. Early changes in phonatory stability, frequency control, and noise components of the voice signal have been reported in ALS patients with otherwise preserved speech intelligibility, suggesting that objective voice analysis may detect subclinical bulbar involvement [4]. Over the past decades, increasing attention has been paid to the potential role of voice and speech analysis as non-invasive biomarkers in ALS. Acoustic studies have documented alterations in parameters such as fundamental frequency, jitter, shimmer, and harmonic-to-noise ratios, reflecting impaired neuromuscular control of phonation. However, results across studies remain heterogeneous, likely due to differences in disease stage, task selection, analytical methods, and cohort characteristics [3]. A recent systematic review highlighted the promise of voice-based approaches for ALS detection and monitoring, including semi-automatic and automatic classification systems, while also emphasizing the limited size of existing cohorts and the restricted set of features commonly analyzed [5]. Recent studies have also explored the role of acoustic voice metrics in differentiating ALS clinical phenotypes and in capturing bulbar involvement, further supporting the potential of voice-based biomarkers in this population [6]. Beyond acoustic descriptors, the complexity and heterogeneity of ALS progression pose major challenges for clinical characterization and prognosis. Widely used clinical scales, such as the ALSFRS-R, provide valuable global assessments but may insufficiently capture region-specific or early functional changes, particularly at the bulbar level. Large-scale analyses have demonstrated that ALS progression and survival are influenced by multidimensional and partially independent factors, underscoring the need for complementary metrics capable of reflecting specific physiological domains [7].

Accordingly, biomechanical analysis of voice production has emerged as a promising approach to bridge the gap between acoustic signal features and underlying laryngeal physiology. By modeling vocal fold vibration dynamics, tissue behavior, and neuromuscular control, biomechanical parameters may provide a more direct representation of the physiological mechanisms affected in ALS. However, evidence on the application of biomechanical voice analysis in ALS remains scarce, particularly regarding its ability to differentiate clinical phenotypes and to relate objective vocal alterations to functional clinical measures. This study addresses this gap by combining acoustic and biomechanical voice analysis, with particular emphasis on biomechanical parameters, with established clinical scales to explore their complementary value in characterizing bulbar involvement and functional status in ALS.

## 2. Materials and Methods

### 2.1. Study Design

A cross-sectional observational study was conducted with the aim of differentiating the biomechanical characteristics of voice in patients with ALS-B and those with ALS-S. The study population also included a control group composed of patients with voice disorders of non-neurological origin, used as a comparative reference to contextualize voice characteristics specifically associated with ALS. In addition, exploratory secondary analyses were conducted to examine the potential clinical relevance of biomechanical voice parameters in relation to established measures of disease severity and functional status, including the ALSFRS-R [8] and the Barthel Index [9].

The study was conducted in accordance with the Declaration of Helsinki and approved by the Ethics Committee of the Virgen Macarena University Hospital in Seville, Spain (Code: PDB-AFELA-2019), approval date is 8 May 2019.Informed consent was obtained from all subjects involved in the study, and the procedures posed no risk to them. Written informed consent has been obtained from the patients to publish this paper.

### 2.2. Patient Selection and Recruitment

A total of fifty patients diagnosed with ALS were consecutively enrolled between July 2019 and September 2024 at the Phoniatrics Unit of Virgen Macarena University Hospital (Seville, Spain). As participants were recruited from a single tertiary referral center, a potential referral bias toward more complex cases cannot be excluded, and the findings should therefore be interpreted with caution. The study population comprised 31 men (62%) and 19 women (38%), with a mean age of 64.14 years (SD ± 8.79; 95% CI: 62.14–65.89). All participants provided written informed consent prior to inclusion in the study.

Sample size estimation was initially performed to assess differences in the prevalence of voice alterations between patients with ALS and the general population. Based on previously reported prevalence rates (80% in ALS and 17% in the general population), a two-proportion comparison using GRANMO software (version 8.0) indicated that a minimum of 10 participants per group was required (α = 0.05; power = 80%), with adjustment for an expected 10% attrition rate. Groups were matched by age and sex to reduce potential confounding. However, this matching should be considered exploratory, as subsequent analyses of biomechanical and acoustic voice parameters were primarily exploratory in nature.

Eligible patients were consecutively identified through medical record review during routine clinical appointments by the same experienced phoniatrician, in order to minimize selection bias. All examinations followed a standardized protocol to ensure methodological consistency and minimize inter-rater variability. The assessment included a structured clinical interview, perceptual voice evaluation using the GRBAS scale [10], and instrumental voice recording procedures. These procedures were based on previously established clinical protocols and adapted for the purposes of the present study.

### 2.3. Inclusion and Exclusion Criteria

Eligibility criteria required a confirmed diagnosis of ALS according to the revised El Escorial criteria, native proficiency in Spanish, and preserved cognitive functioning, defined as a Montreal Cognitive Assessment (MoCA) score of 26 or higher [11]. The mean disease duration at the time of evaluation was 32.18 months (SD ± 32.86; 95% CI: 22.84–41.52). When stratified by clinical phenotype, patients with ALS-S presented a mean disease duration of 30.70 months (SD ± 27.60; 95% CI: 20.40–41.00), whereas those with ALS-B showed a mean duration of 34.40 months (SD ± 40.20; 95% CI: 15.50–53.20).

Patients with ALS presenting concomitant neurological conditions or a history of non-neurological voice disorders were excluded from the study.

The non-ALS comparison group consisted of patients attending a specialized voice clinic for dysphonia, with no diagnosis of neurological disease. All control participants reported self-perceived voice impairment, with Voice Handicap Index-10 (VHI-10) scores > 10. Controls were selected at random from eligible clinical cases. Laryngological diagnoses in the non-ALS group included functional dysphonia (26.9%), inflammatory dysphonia (21.2%), laryngopharyngeal reflux-related dysphonia (19.7%), vocal nodules (10.9%), vocal atrophy (11.9%), Reinke’s edema (6.5%), leukoplakia (1.5%), unilateral vocal fold paralysis (0.7%), and intracordal cyst (0.7%).

Information on ongoing or prior dysphonia treatments was not systematically available and is acknowledged as a limitation.

### 2.4. Voice Recording and Signal Processing

Voice recordings were obtained by asking participants to sustain the vowel /a/ for several seconds under controlled conditions. Only samples with a minimum stable phonation of three seconds were retained for analysis, allowing extraction of a representative mid-portion of the signal. Recordings were performed using a unidirectional Saramonic SR-XM1 microphone (Saramonic International, Shenzhen, China), positioned at a fixed distance of approximately 20–30 cm from the participant’s mouth.

All recordings were conducted in a quiet clinical environment. The clinician-initiated recording once a stable phonation had been achieved, thereby minimizing onset and offset artifacts. Input amplitude was continuously monitored via the recording interface and maintained within 30–80% of the available dynamic range to avoid clipping or under-modulation. No post hoc amplitude normalization was applied prior to signal analysis. Signal analysis was performed without access to information on the clinical phenotype.

Biomechanical voice parameters were subsequently extracted using the Voice Clinical Systems^®^ mobile application (version 1.4.0) [12], which generates a standardized R3 report comprising 22 biomechanical variables related to vocal fold vibration, tissue dynamics, and neuromuscular control. All biomechanical parameters included in the R3 report are defined and described in detail in Table 1.

Raw output values generated by the Voice Clinical Systems^®^ software were used for all statistical analyses.

Acoustic analysis was performed using the Praat software package (version 6.2.22) [13]. For each participant, a stable three-second segment of sustained phonation was selected for analysis. This segment corresponded to the most stationary portion of the vocal signal and was extracted from broadband spectrograms. The acoustic parameters obtained included fundamental frequency (F_0_), jitter, shimmer, and the harmonics-to-noise ratio (HNR), which together provide quantitative information regarding vocal stability, periodicity, and overall voice quality (Table 2). Intra-rater reliability was assessed using a randomly selected subset of recordings and showed acceptable agreement.

### 2.5. Perceptual Voice Assessment and Functional Measures

Perceptual evaluation of voice quality was conducted using the GRBAS scale, which assesses five auditory-perceptual dimensions: Grade, Roughness, Breathiness, Asthenia, and Strain. Each parameter was rated according to standardized criteria, allowing a structured clinical assessment of vocal impairment (Table 3).

During the same assessment session, participants were additionally evaluated using two functional scales. The Barthel Index was employed to quantify independence in activities of daily living, encompassing domains such as feeding, personal hygiene, mobility, transfers, and stair climbing, with scores ranging from 0 (complete dependence) to 100 (full independence). In parallel, functional impairment related to amyotrophic lateral sclerosis was assessed using the ALS Functional Rating Scale–Revised (ALSFRS-R), which evaluates bulbar, motor, and respiratory function (Table 4).

In addition, a bulbar ALSFRS-R subscore was calculated as the sum of items 1–3 (speech, salivation, swallowing; range 0–12) to characterize bulbar functional status at the time of voice recording.

### 2.6. Data Analysis

Statistical analyses were performed using R (version 4.0.5) [14] and SPSS (version 29.0.2) [15]. Data distribution and homogeneity of variances were assessed using the Shapiro–Wilk and Levene tests, respectively. As most variables did not meet normality assumptions, non-parametric statistical tests were applied. Continuous variables are therefore presented as median and interquartile range (IQR). For descriptive purposes, selected demographic variables are presented as mean ± standard deviation (SD). Given the exploratory nature of this cross-sectional study, no single primary endpoint was pre-specified and the analyses should be interpreted as hypothesis-generating. Accordingly, group comparisons were performed using the Mann–Whitney U test or the Kruskal–Wallis test, as appropriate. Effect sizes were calculated for the main non-parametric between-group comparisons using epsilon-squared (ε^2^) to facilitate clinical interpretation.

Associations between acoustic and biomechanical voice parameters and clinical severity were explored by comparing variables across categories of perceptual voice severity (GRBAS), stratified levels of ALSFRS-R and functional independence (Barthel Index). These comparisons were conducted using non-parametric tests due to non-normal data distribution.

Post hoc pairwise comparisons following the Kruskal–Wallis test were performed using Dunn’s test with Benjamini–Hochberg false discovery rate (FDR) correction, to reduce the risk of type I error associated with multiple pairwise comparisons. For two-group comparisons, the Mann–Whitney U test was used. Categorical variables were analyzed using the chi-square or Fisher’s exact test. For parameters known to be sex-dependent, analyses were additionally stratified by sex. Statistical significance was set at *p* < 0.05.

Adjusted multivariable linear regression models with robust standard errors were fitted to assess the robustness of key biomechanical findings while accounting for potential confounding. Outcomes were log(1 + x)-transformed due to skewness and clinically relevant covariates were selected a priori (age, sex, smoking status, and disease duration when appropriate). Model assumptions were assessed through residual inspection and variance inflation factors.

## 3. Results

### 3.1. Sociodemographic and General Characteristics

The study sample consisted of a total of 100 participants: 50 non-ALS patients with non-neurological dysphonia (non-ALS group), 20 with ALS-B, and 30 with spinal-onset ALS-S. The demographic characteristics of the study population are summarized in Table 5. No relevant differences in age distribution were observed between the ALS and non-ALS groups, supporting comparability between cohorts prior to the analysis of vocal parameters.

The distribution of sex did not differ significantly between groups (*p* > 0.05). Similarly, no significant differences were observed in smoking status between ALS and non-ALS participants.

Among patients with ALS, disease duration did not differ significantly between bulbar and spinal phenotypes. Descriptive statistics for demographic and clinical variables are summarized in Table 6.

### 3.2. Discriminative Acoustic and Biomechanical Parameters Between ALS and Non-ALS Groups

Consistent with the use of non-parametric statistical tests, median and interquartile range (IQR) values are reported below for variables showing statistically significant differences; median (IQR) values for all remaining acoustic and biomechanical parameters are provided in Supplementary Table S1.

In the analysis of the acoustic parameters, statistically significant differences were observed between the ALS and Non-ALS groups only for the fundamental frequency (F_0_) and the shimmer parameter (Table 7). Accordingly, F0 showed a higher median value in ALS (173.81 [134.50–196.97]) than in controls (134.65 [111.35–195.03]), and shimmer was also higher in ALS (5.61 [3.85–7.84]) than in controls (4.34 [2.81–5.93]). No significant differences were identified in the remaining acoustic variables evaluated.

Regarding the biomechanical parameters, significant differences were detected between the ALS and Non-ALS groups in the parameters Pr1, Pr11, Pr12 and Pr14 (Table 7). The between-group difference observed for Pr14 showed a moderate effect size (ε^2^ = 0.10), supporting the clinical relevance of this finding.

To evaluate the robustness of the observed between-group difference in Pr14, a multivariable linear regression model was fitted including age, sex, and smoking status as covariates. After adjustment, ALS status remained independently associated with higher Pr14 values (β = 0.77, 95% CI 0.31–1.24; *p* = 0.001), supporting that the observed alteration was not explained by these potential confounders (Supplementary Table S2).

**Figure 1:**
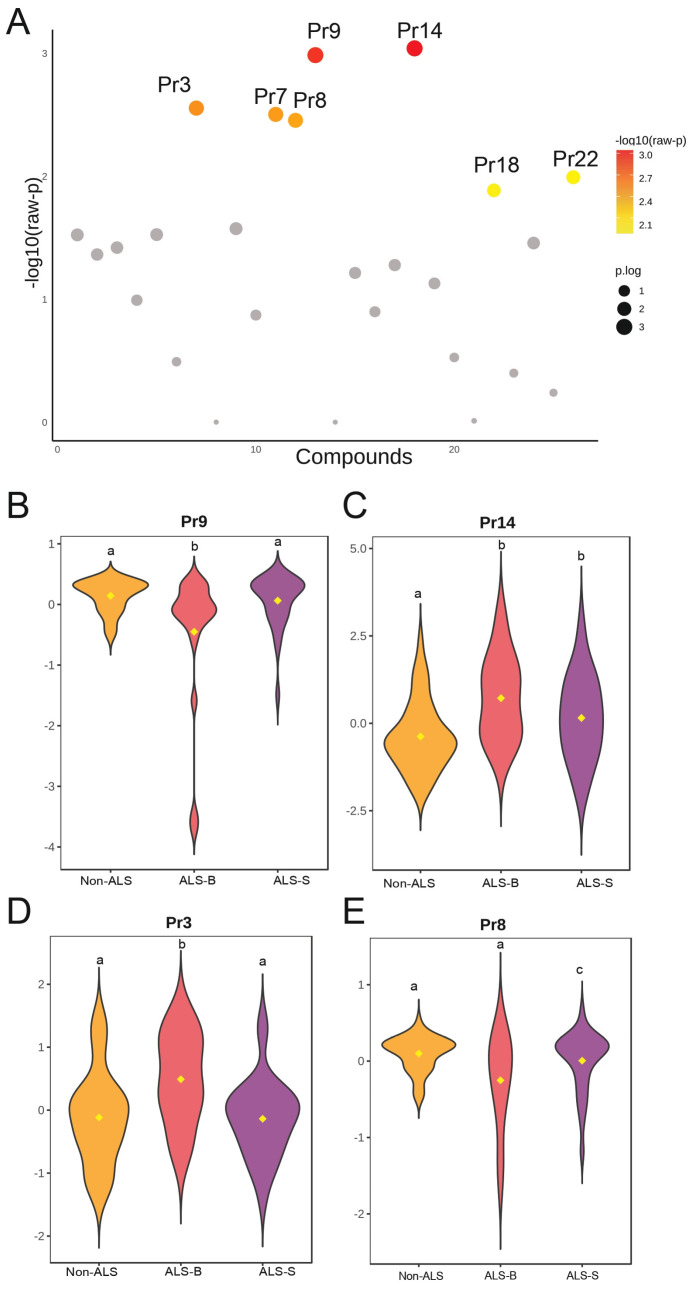
Differential analysis of phonetic parameters across study groups. (**A**) Overview of group differences for all analyzed phonetic parameters. Each point represents a parameter plotted by its—log10(raw *p*-value) from the Kruskal–Wallis test; color (yellow–red) denotes statistical significance and size reflects effect strength. Parameters with the strongest group effects are labeled. (**B**–**E**) Violin plots illustrate the distribution of selected parameters (Pr9, Pr14, Pr3, Pr8) across Non-ALS, bulbar-onset ALS (ALS-B), and spinal-onset ALS (ALS-S) groups. Differences were evaluated using the Kruskal–Wallis test followed by Dunn’s post hoc test with Benjamini–Hochberg correction. Groups sharing a letter are not significantly different, whereas different letters indicate *p* < 0.05.

In a complementary analysis, the Kruskal–Wallis test identified significant between-group differences in seven biomechanical parameters (Pr3, Pr7, Pr8, Pr9, Pr14, Pr18 and Pr22) (*p* < 0.05). Among these, the most consistent phenotype-related differences were observed for Pr3, Pr8, Pr9 and Pr14 in pairwise comparisons between ALS-B and ALS-S, indicating distinct biomechanical patterns of vocal fold behavior associated with each clinical phenotype. These four parameters are presented in detail in Table 8 and Figure 1.

### 3.3. Clinical and Functional Differences Between ALS-B and ALS-S

**Figure 2:**
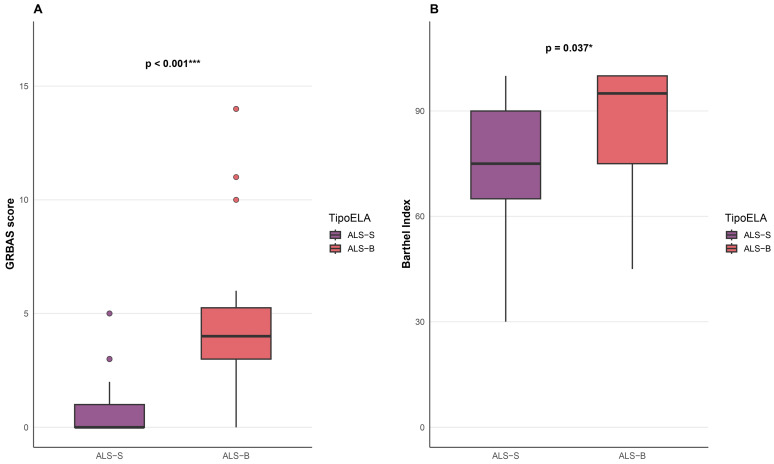
Comparison of GRBAS score (**A**) and Barthel Index (**B**) between spinal-onset (ALS-S) and bulbar-onset (ALS-B) amyotrophic lateral sclerosis patients. Boxplots show the median, interquartile range, whiskers extending to 1.5 × IQR, and outliers. Dots represent individual participant values. Group differences were analyzed using the Wilcoxon rank-sum test (Mann–Whitney U). GRBAS scores were significantly higher in ALS-B (*p* < 0.001), indicating greater voice impairment, while the Barthel Index also differed significantly (*p* = 0.037), reflecting reduced functional independence in spinal-onset patients compared to bulbar-onset patients. * *p* < 0.05; *** *p* < 0.001.

Regarding clinical and functional measures, significant differences between ALS-B and ALS-S were observed for perceptual voice severity (GRBAS) and functional independence, as assessed by the Barthel Index. Patients with ALS-B showed higher GRBAS scores and higher Barthel Index values compared with those with ALS-S (Table 9, Figure 2). In contrast, no significant differences were observed between subgroups for global functional status as measured by the ALSFRS-R, nor for other clinical variables. To provide clinical context regarding bulbar status at the time of voice recording, we additionally analyzed the ALSFRS-R bulbar subscore (items 1–3). Patients with ALS-B showed lower bulbar subscores than those with ALS-S (median 9.0 [IQR 6.75–10.0] vs. 11.5 [IQR 10.0–12.0]), indicating greater bulbar functional impairment at assessment.

Overall, ALS-B was associated with greater perceptual voice impairment, while functional independence (Barthel Index) was relatively preserved in this cohort compared with ALS-S. These associations represent descriptive trends and should not be interpreted as causal or predictive.

In a multivariable model restricted to ALS patients and including phenotype, age, sex, disease duration, and bulbar ALSFRS-R subscore, the association between phenotype and Pr8 was attenuated and no longer statistically significant (β = −0.69, 95% CI −1.80 to 0.41; *p* = 0.219). In contrast, disease duration remained independently associated with the parameter (β = −0.015, 95% CI −0.026 to −0.003; *p* = 0.017) (Supplementary Table S3). These findings suggest that part of the observed biomechanical variability may reflect current functional status rather than phenotype alone.

**Figure 3:**
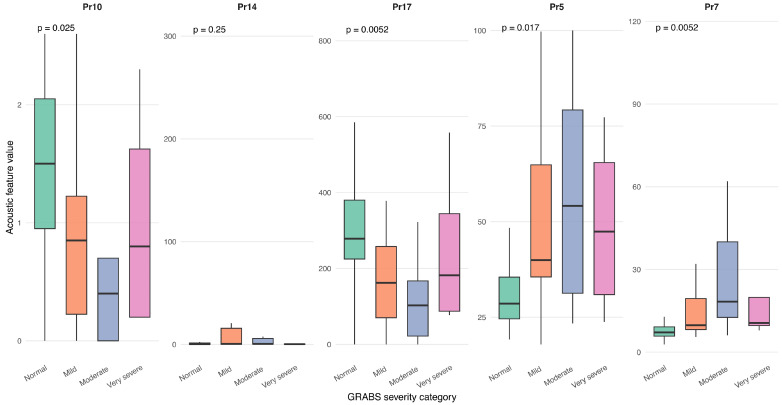
Boxplots showing the distribution of selected acoustic voice parameters (Pr10, Pr14, Pr17, Pr5, Pr7) across GRBAS severity categories in patients with amyotrophic lateral sclerosis (ALS). Panels display the median, interquartile range, whiskers (1.5 × IQR), and outliers for the Normal, Mild, Moderate, and Very severe groups. Differences were analyzed using the Kruskal–Wallis test with multiple-comparison adjustment. Significant effects were observed for Pr10 (*p* = 0.031), Pr17 (*p* = 0.013), Pr5 (*p* = 0.028), and Pr7 (*p* = 0.013), whereas Pr14 was not significant (*p* = 0.249). Overall, acoustic parameters varied with voice quality severity, except for Pr14. an association between increasing perceptual impairment and altered biomechanical vocal fold behavior.

Additional analyses explored the relationship between voice parameters and clinical severity measures. No statistically significant associations were observed between acoustic parameters and the clinical severity measures evaluated. Several biomechanical voice parameters varied significantly across levels of perceptual voice impairment as assessed by the GRBAS scale. Specifically, parameters Pr4, Pr5, Pr7, Pr10 and Pr17 showed significant differences according to GRBAS severity categories, with higher median values and greater dispersion observed in patients with more severe dysphonia (Figure 3).

**Figure 4:**
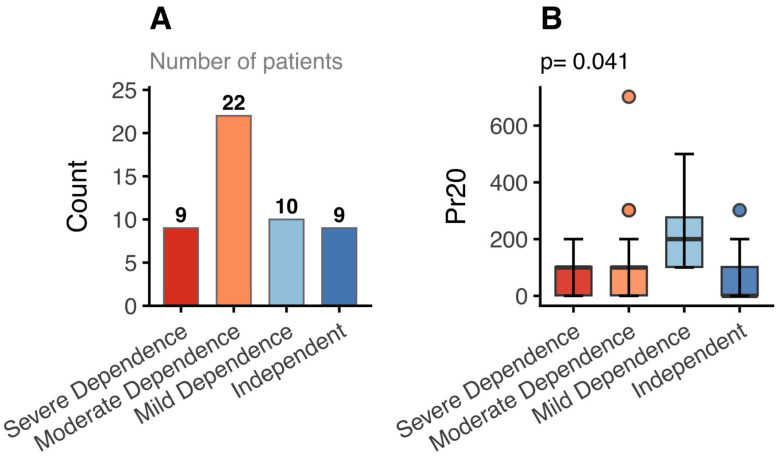
Distribution of Pr20 across Barthel Index functional categories in patients with amyotrophic lateral sclerosis (ALS). (**A**) Number of patients within each functional dependence category. (**B**) Boxplots showing the distribution of Pr20 values across Barthel categories. Boxes represent the median and interquartile range, whiskers extend to 1.5 × IQR, and points indicate outliers. Differences between groups were assessed using the Kruskal–Wallis test (*p* = 0.041).

Regarding global functional status, only parameter Pr15 showed significant differences across functional categories defined by the ALSFRS-R (*p* < 0.05). When functional status was stratified using the Barthel Index, two biomechanical parameters (Pr15 and Pr20) differed significantly between groups. However, because Pr15 typically presents values close to zero under normal conditions, its group differences were not visually distinguishable in the plots. Therefore, Figure 4 displays only the distribution of Pr20 across Barthel categories, showing higher and more variable values in patients with lower Barthel scores (*p* < 0.05).

## 4. Discussion

### 4.1. Acoustic Voice Features in ALS

In the present cohort, acoustic analysis revealed significant differences between ALS and non-ALS participants in fundamental frequency (F_0_) and shimmer. An increase in F_0_ was more pronounced in the ALS-B, whereas shimmer values were higher in ALS-S. However, no statistically significant differences were observed between ALS subtypes, suggesting that while acoustic measures are sensitive to disease-related phonatory alterations, their discriminatory capacity within ALS phenotypes remains limited in this sample.

These findings partially contrast with previous reports. A meta-analysis by Chiaramonte and Bonfiglio (2019) [16] described increased jitter and shimmer in ALS-B, with no consistent changes in F_0_ or HNR compared with controls. Differences in task selection, inclusion criteria, and acoustic metrics may account for these discrepancies [3]. Classical studies and reviews have consistently reported increased jitter and shimmer and reduced HNR in ALS, even in perceptually normal voices [4], whereas results regarding F_0_ have been heterogeneous, with occasional elevations in bulbar phenotypes or sex-specific subgroups. In this context, shimmer appears to be sensitive to amplitude instability, while F_0_ elevation in bulbar ALS may reflect compensatory increases in glottal tension secondary to neuromuscular impairment [3].

Recent studies using automatic and machine-learning–based approaches support the relevance of acoustic features for detecting bulbar involvement and estimating disease severity. Simmatis et al. (2024) [17] demonstrated good discrimination between ALS and controls using a compact acoustic feature set, while Dubbioso et al. (2024) [18] identified acoustic markers associated with dysarthria severity. Overall, our findings suggest that acoustic analysis provides useful contextual information, particularly in ALS-B, but its isolated use offers only moderate discrimination and remains highly dependent on acquisition conditions and inter-speaker variability, as also suggested by Tena et al. (2022) [19]. In contrast to our preliminary study [20], no consistent acoustic differences were observed here, possibly due to the limited sample size of the earlier cohort.

### 4.2. Biomechanical Voice Analysis and Clinical Integration

Beyond acoustic measures, biomechanical voice analysis represents the central contribution of this study, as it enables a closer approximation to laryngeal physiology and neuromuscular involvement. Significant differences between ALS and non-ALS participants were observed in biomechanical parameters reflecting complementary aspects of vocal fold function, including Pr1 (related to fundamental frequency regulation), Pr11 and Pr12 (associated with incomplete or irregular glottal closure), and Pr14 (reflecting cycle-to-cycle vibratory instability). These alterations indicate impaired coordination of vocal fold vibration and closure mechanisms in ALS. Importantly, the between-group difference observed for Pr14 remained significant after adjustment for age, sex, and smoking status, reinforcing the robustness of this biomechanical alteration.

Differences between ALS-B and ALS-S further highlight the sensitivity of biomechanical parameters to differences in bulbar motor involvement at the time of assessment across clinical trajectories. These differences may partly reflect the current functional burden rather than phenotype-specific mechanisms alone; accordingly, functional status should be considered when interpreting voice alterations in ALS. Parameters reflecting vibratory asymmetry (Pr3) and cycle-to-cycle vibratory instability (Pr14) were higher in ALS-B, whereas parameters reflecting increased tension and force during glottal closure (Pr8 and Pr9) were higher in ALS-S, which may reflect increased laryngeal closure effort/compensatory hyperfunction in the spinal-onset phenotype. Conversely, adjusted analyses within the ALS cohort showed that disease duration, rather than phenotype, remained independently associated with Pr8, suggesting that part of the observed biomechanical variability may reflect temporal disease burden rather than phenotype-specific mechanisms alone.

These findings align with previous work supporting the validity of biomechanical voice analysis as an objective assessment tool. Gómez-Vilda et al. (2007) [21] demonstrated that biomechanical parameters can detect vocal fold dysfunction even in the absence of overt acoustic abnormalities, while Cardoso et al. (2022) [12] confirmed their sensitivity in functional and organic dysphonias. Similar biomechanical alterations have been reported in other neurological conditions, including Parkinson’s disease and multiple sclerosis [22], further supports the view that biomechanical voice analysis captures the vocal imprint of neuromuscular disorders. However, validation studies specifically conducted in ALS populations remain limited; therefore, these findings should be interpreted within an exploratory framework pending further disease-specific reliability studies.

The integration of biomechanical parameters with clinical scales provides a more comprehensive view of disease impact. Higher GRBAS scores were associated with alterations in parameters such as Pr4, Pr5, and Pr7 (related to the temporal organization of the closing approach, the duration of vocal fold separation, and the opening phase of the vibratory cycle) suggesting that increasing perceptual severity of dysphonia corresponds to prolonged and inefficient vibratory patterns. These objective alterations may support the potential clinical relevance of biomechanical voice analysis as a complementary marker of bulbar dysfunction.

Similarly, reduced functional independence, as measured by the Barthel Index, was associated with variations in parameters such as Pr15, reflecting vibratory blocking events, and Pr20, related to tissue changes consistent with edema during the opening phase. These associations suggest that biomechanical voice alterations may reflect broader clinical status rather than representing isolated phenomena. The lack of significant differences between ALS phenotypes in ALSFRS-R scores is consistent with the global nature of this scale, which may obscure domain-specific deficits in phonation and communication, supporting the use of complementary instruments such as GRBAS and the Barthel Index [1,8,23,24].

Overall, these findings support biomechanical voice analysis as a sensitive, non-invasive approach for capturing clinically relevant aspects of bulbar involvement and functional status in ALS, although results should be interpreted considering disease heterogeneity and sample size limitations. Given the exploratory nature of the study and the large number of biomechanical parameters evaluated, effect sizes selectively reported for the principal between-group comparisons to support clinical interpretation. In addition, the modest sample size for multivariable analyses may have limited statistical power. Accordingly, the findings should be interpreted as hypothesis-generating. The absence of a normophonic control group warrants interpreting the present findings primarily as hypothesis-generating rather than definitive evidence of disease-specific alterations. Furthermore, the restriction of the study population to patients with preserved cognitive function may limit the generalizability of these findings to ALS patients with cognitive impairment. Larger, longitudinal and multicenter studies are warranted to confirm these associations and to explore their prognostic value. Future research should also further evaluate the reliability and reproducibility of biomechanical measurements derived from the Voice Clinical Systems^®^ platform, particularly within ALS populations, to support their broader clinical applicability.

## 5. Conclusions

The robustness of key biomechanical findings is further supported by adjusted analyses accounting for relevant demographic variables. This study demonstrates that biomechanical voice analysis suggests potential utility for capturing physiologically meaningful aspects of vocal impairment in ALS, complementing conventional acoustic measures and clinical scales. While acoustic features offer contextual insight, biomechanical parameters more effectively capture alterations associated with bulbar motor involvement, particularly in ALS-B. The integration of biomechanical voice metrics with perceptual and functional assessments enhances characterization of bulbar dysfunction and overall clinical status. These findings highlight the promise of biomechanical voice analysis as a non-invasive candidate marker that may contribute to the clinical characterization of ALS. Future longitudinal studies are required to validate its prognostic value and clinical applicability.

## Figures and Tables

**Table 1:** Biomechanical voice parameters (Pr1–Pr22): Definition/clinical meaning and reference values.

Parameter	Measure	Definition and Clinical Meaning (Merged)	Reference Range in Normophonic Voices (♀/♂)
Pr1	Fundamental frequency (F_0_)	Measures the baseline vibratory rate; reflects pitch regulation and vibratory stability	180–240 Hz/105–139 Hz
Pr2	Proportion of cycles with closure predominance	Quantifies the predominance of the closing/adductory pattern across cycles; reflects closure regularity	1 VFa/VFb
Pr3	Increase in open phase/asymmetry	Captures increased opening and/or hemicycle imbalance; suggests asymmetric vibration or instability	0%
Pr4	Time in the approach-to-closure phase	Percentage spent moving toward closure; reflects coordination/efficiency of the glottal gesture	55–70%/50–73%
Pr5	Time with vocal folds separated	Percentage of time without contact; associated with reduced contact and greater airflow escape	30–45%/26–49%
Pr6–Pr7	Duration of the opening phase	Describes the temporal weight of the opening component; informs airflow control and phonatory coordination	30–35%/12–27%; 8–11%/5–36%
Pr8	Tension during glottal closure	Estimates closure-related tension; higher values are compatible with hyperfunctional/effortful phonation	1–26 r.u/1.5–13 r.u
Pr9	Glottal closure force	Estimates closure intensity; may indicate compensatory behavior or inefficient phonation	80–749 r.u/95–799 r.u
Pr10	Optimal energy use index	Summarizes phonatory energy efficiency; higher values typically indicate more efficient/sustainable phonation	1.2–1.7 r.u/1.2–1.6 r.u
Pr11–Pr12	Incomplete or irregular closure	Detects leakage due to incomplete/unstable closure; consistent with increased air escape	0 r.u; 0 r.u
Pr13–Pr14	Cycle-to-cycle vibratory instability	Quantifies inter-cycle variability; elevated values are consistent with impaired neuromotor control/tremor	<8 r.u/<17 r.u; 0 r.u
Pr15	Vibratory blocking events	Counts interruptions/blocks during vibration; suggests spasmodic patterns or marked irregularity	0 r.u
Pr16	Glottal gap width	Estimates the relative glottal gap amplitude; linked to glottal configuration and closure pattern	0.2–1.1 r.u/0.25–1.5 r.u
Pr17–Pr18	Mucosal wave correlation (closure/opening)	Quantifies coherence of mucosal wave behavior; reflects tissue pliability/elastic vibratory behavior	190–330/170–520 r.u; 20–65/15–89 r.u
Pr19–Pr20	Edema correlation (closure/opening)	Captures signals compatible with tissue load/edema; often increases with higher vocal load/effort	(−10)–60/(−18)–54 r.u; 0–100 r.u
Pr21	Structural imbalance index	Summarizes asymmetry/rigidity; higher values suggest increased stiffness or laryngeal asymmetries	<75 r.u
Pr22	Structural mass/supraglottic alteration index	Signals patterns compatible with structural lesion or supraglottic hyperfunction	0 r.u

Note: Hz: hertz; VFa/VFb: Ratio of formant velocities or vocal frequencies between conditions A and B. As this represents a ratio between homogeneous magnitudes, it is expressed without units (relative value); r.u.: relative units provided by the Voice Clinical Systems^®^ software; F/M: female/male.

**Table 2:** Acoustic parameters.

Parameter	Description	Reference Range (♀/♂)
Fundamental frequency (F_0_)	Mean fundamental frequency during sustained phonation	180–240 Hz/105–139 Hz
Shimmer (%)	Amplitude perturbation	<3%
Jitter (%)	Frequency perturbation	<1%
Harmonics-to-noise ratio (HNR)	Harmonic-to-noise energy ratio	>20 dB

Notes: Hz = Hertz; dB = decibels.

**Table 3:** GRBAS perceptual voice assessment.

Parameter	Component Assessed	Scoring
G (Grade)	Overall voice quality	0–3
R (Roughness)	Irregularity of vocal fold vibration	0–3
B (Breathiness)	Audible air leakage	0–3
A (Asthenia)	Vocal weakness	0–3
S (Strain)	Excessive vocal effort	0–3

Note: Dysphonia severity was categorized for descriptive purposes as mild (0–3), moderate (4–6), severe (7–9), and very severe (10–15).

**Table 4:** ALSFRS-R functional domains.

Functional Domain	Included Items	Scoring
Bulbar function	Speech, salivation, swallowing	0–12 (items 1–3; each item scored 0–4)
Fine motor function	Writing, handling utensils, dressing	0–4
Gross motor function	Turning in bed, walking, climbing stairs	0–4
Respiratory function	Dyspnea, orthopnea, ventilatory support	0–4

Note: The ALSFRS-R total score ranges from 0 to 48, with higher scores indicating better functional status. The bulbar subscore corresponds to the sum of items 1–3 (speech, salivation, swallowing) and ranges from 0 to 12, with higher scores indicating better bulbar function.

**Table 5:** Sociodemographic characteristics.

Group	Mean Age ± SD (Years)	95% CI
No-ALS	64.16 ± 7.72	61.96–66.84
ALS (total)	64.14 ± 9.83	61.35–66.93
ALS-B	67.05 ± 10.59	63.33–70.77
ALS-S	62.20 ± 10.59	58.25–66.15
Total sample	64.15 ± 8.79	62.14–65.89

Notes: ALS-B: bulbar ALS. ALS-S: spinal ALS. Values are expressed as mean ± standard deviation (SD). CI: confidence interval.

**Table 6:** Demographic and clinical characteristics of the study population.

Variable	No-ALS	ALS-B	ALS-S
Sex (male/female)	31/19	10/10	21/9
Smoking status	10	8	12
Disease duration, months (mean ± SD)	Non applicable	34.40 ± 40.20	30.70 ± 27.60

**Table 7:** Acoustic and biomechanical parameters showing significant differences between ALS and non-ALS groups.

Parameters	Non-ALS (Median [IQR])	ALS (Median [IQR])	*p*-Value
F_0_ (Hz)	134.65 [111.35–195.03]	173.81 [134.50–196.97]	0.026
Shimmer (%)	4.34 [2.81–5.93]	5.61 [3.85–7.84]	0.018
Pr1	135.05 [111.75–195.00]	172.95 [134.95–196.45]	0.029
Pr11	−0.01 [−0.05–0.00]	0.00 [−0.01–0.00]	0.016
Pr12	16.55 [0.00–32.08]	0.00 [0.00–9.68]	0.009
Pr14	0.000 [0.000–0.427]	0.523 [0.000–4.921]	<0.001

Notes: Values are expressed as median (IQR). Only statistically significant parameters are shown. Statistical significance was set at *p* < 0.05.

**Table 8:** Biomechanical voice parameters showing significant differences between bulbar- and spinal-onset ALS.

Parameters	ALS-B (Median [IQR])	ALS-S (Median [IQR])	*p*-Value
Pr3	0.00 [0.00–69.68]	0.00 [0.00–0.00]	0.037
Pr8	11.90 [4.53–41.65]	40.15 [9.30–164.82]	0.023
Pr9	689.00 [298.93–1744.55]	2743.65 [509.85–12,173.75]	0.028
Pr14	1.10 [0.00–7.65]	0.490 [0.062–2.258]	0.004

**Table 9:** Clinical and Functional Measures Showing Significant Differences Between ALS-B and ALS-S.

Parameters	ALS-B (Median [IQR])	ALS-S (Median [IQR])	*p*-Value
GRBAS	4.0 [3.00–5.25]	0.00 [0.00–1.00]	<0.001
Barthel Index	95.00 [75.00–100.00]	75.00 [65.00–90.00]	0.037

## Data Availability

The data presented in this study are available on request from the corresponding author, because the data are not publicly available due to privacy and ethical restrictions.

## Supplementary Materials

The following supporting information can be downloaded at: https://www.mdpi.com/article/10.3390/medsci14010112/s1, Table S1: Median (IQR) values of acoustic and biomecanichal voice parameters by group; Table S2: Adjusted multivariable linear regression model for Pr14 comparing ALS and non-ALS participants (robust HC3 standard errors); Table S3: Adjusted multivariable linear regression model for log (1+Pr8) within ALS participants (robust HC3 standard errors).

## Abbreviations

The following abbreviations are used in this manuscript:

ALS	Amyotrophic lateral sclerosis
ALS-B	Bulbar-onset amyotrophic lateral sclerosis
ALS-S	Spinal-onset amyotrophic lateral sclerosis
ALSFRS-R	Amyotrophic Lateral Sclerosis Functional Rating Scale-Revised
CI	Confidence interval
F_0_	Fundamental frequency
GRBAS	Grade, Roughness, Breathiness, Asthenia, Strain
HNR	Harmonics-to-noise ratio
MoCA	Montreal Cognitive Assessment
Non-ALS	Participants without amyotrophic lateral sclerosis
Pr1–Pr22	Biomechanical parameters
SD	Standard deviation
