# Supplementary material for: Biomechanical Voice Parameters as Potential Biomarkers for Phenotype Differentiation in Amyotrophic Lateral Sclerosis: A Cross-Sectional Study
Source: Med Sci (Basel). 2026 Feb 26;14(1):112. doi: 10.3390/medsci14010112 (PMC13027408; doi:10.3390/medsci14010112)
Supplement: Supplementary file 1 [file medsci-14-00112-s001.zip › medsci-4113899-supplementary.pdf]

Table S1. *Median (IQR) values of acoustic and biomechanical voice parameters by group*

Parameters	ALS- total	ALS- B	ALS- S	Control
F_0 (Hz)	173.81 [134.50–196.97]	180.07 [133.42–200.82]	167.51 [141.23–196.98]	134.65 [111.35–195.03]
Jitter (%)	0.387 [0.283–0.509]	0.43 [0.34–0.54]	0.33 [0.26–0.46]	0.36 [0.27–0.62]
Shimmer (%)	5.61 [3.85–7.84]	5.76 [3.96–7.60]	5.18 [3.67–7.84]	4.34 [2.81–5.93]
HNR (Db)	16.74 [13.64–19.78]	16.51 [13.66–18.11]	17.49 [13.73–20.56]	17.92 [15.49–21.18]
Pr1	172.95 [134.95–196.45]	180.40 [133.60–198.83]	168.65 [140.70–196.45]	135.05 [111.75–195.00]
Pr2	1.00 [1.00–1.00]	1.00 [0.50–1.00]	1.00 [1.00–1.00]	1.00 [1.00–1.00]
Pr3	0.00 [0.00–54.90]	0.00 [0.00–69.68]	0.00 [0.00–0.00]	0.00 [0.00–0.00]
Pr4	64.10 [42.10–72.55]	45.85 [34.55–69.20]	67.00 [55.30–72.55]	60.75 [51.45–69.55]
Pr5	35.90 [27.45–57.90]	54.15 [30.80–65.45]	33.00 [27.45–44.70]	39.25 [30.45–48.55]
Pr6	24.45 [16.60–30.88]	26.85 [17.18–34.95]	22.40 [16.60–29.05]	28.05 [19.60–37.00]
Pr7	9.50 [7.00–16.88]	12.20 [8.78–35.20]	8.55 [6.72–12.53]	9.80 [6.90–12.40]
Pr8	20.60 [5.75–123.42]	11.90 [4.53–41.65]	40.15 [9.30–164.82]	50.70 [11.70–148.80]
Pr9	1048.40 [337.68–8078.57]	689.00 [298.93–1744.55]	2743.65 [509.85–12173.75]	2759.70 [681.02–9924.02]
Pr10	1.00 [0.30–1.75]	0.400 [0.150–1.575]	1.15 [0.72–1.75]	1.00 [0.50–1.30]
Pr11	0.00 [-0.01–0.00]	0.00 [-0.01–0.00]	0.00 [-0.01–0.00]	-0.01 [-0.05–0.00]
Pr12	0.00 [0.00–9.68]	0.00 [0.00–4.53]	0.00 [0.00–9.68]	16.55 [0.00–32.08]
Pr13	5.05 [1.32–10.78]	5.85 [3.33–10.90]	3.50 [0.85–10.30]	7.20 [0.55–20.15]
Pr14	0.523 [0.000–4.921]	1.10 [0.00–7.65]	0.490 [0.062–2.258]	0.000 [0.000–0.427]
Pr15	0.00 [0.00–0.00]	0.00 [0.00–0.00]	0.00 [0.00–0.00]	0.00 [0.00–0.00]
Pr16	1.95 [0.50–4.60]	0.75 [0.45–3.02]	2.70 [1.02–6.00]	2.15 [1.02–4.65]
Pr17	215.10 [91.70–316.92]	107.05 [68.80–281.30]	240.75 [153.95–321.82]	221.35 [109.15–309.73]
Pr18	70.45 [29.38–151.70]	92.10 [63.07–213.22]	58.10 [28.45–80.97]	52.45 [40.95–94.10]
Pr19	0.00 [-40.00–12.45]	0.00 [-60.00–33.30]	0.00 [-40.00–0.00]	-20.00 [-38.95–18.75]
Pr20	100.00 [0.00–200.00]	100.00 [0.00–200.00]	100.00 [0.00–100.00]	100.00 [0.00–100.00]
Pr21	95.00 [72.05–95.00]	92.50 [47.45–95.00]	95.00 [77.03–95.00]	95.00 [66.85–95.00]
Pr22	0.00 [0.00–0.00]	0.00 [0.00–0.00]	0.00 [0.00–0.00]	0.00 [0.00–0.00]

**Note: Values are expressed as median [interquartile range].*

Table S1 (1).docx   Table S3.docx   Table S2.docx

Table S2. *Adjusted multivariable linear regression model for Pr14 comparing ALS and non-ALS participants (robust HC3 standard errors).*

**Predictor**	**β**	**95% CI**	***p*-value**
ALS (vs non-ALS)	0.77	0.31 to 1.24	<0.001
Age (years)	0.03	0.00 to 0.05	0.047
Male sex (vs female)	-0.44	-0.93 to 0.06	0.083
Current smoker (vs non-somoker)	-0.09	-0.54 to 0.36	0.708

**Notes: n = 50 ALS participants and n = 50 controls. The outcome was log(1+x)-transformed to account for skewness. Adjusted multivariable linear regression models were fitted with robust standard errors (HC3). Covariates were selected a priori based on clinical plausibility and included age, sex, smoking status, and disease duration when appropriate. ALS status was coded as ALS vs non-ALS; male sex as male vs female; and smoking status as current smoker vs non-smoker.*

Table S3. *Adjusted multivariable linear regression model for log(1+Pr8) within ALS participants (robust HC3 standard errors).*

**Predictor**	**β**	**95% CI**	***p*-value**
Bulbar phenotype (vs spinal)	-0.69	-1.80 to 0.41	0.219
Age (years)	0.03	-0.04 to 0.11	0.333
Male sex (vs female)	0.70	-0.64 to 2.03	0.305
Disease duration (months)	-0.01	-0.03 to 0.00	0.017
ALSFRS-R bulbar subscore (0–12)	0.14	-0.07 to 0.35	0.200

**Notes: n = 50 ALS participants (20 bulbar-onset and 30 spinal-onset). The outcome was log(1+x)-transformed to account for skewness. Adjusted multivariable linear regression models were fitted with robust standard errors (HC3). Covariates were selected a priori based on clinical plausibility. Phenotype was coded as bulbar-onset vs spinal-onset ALS. Disease duration was analyzed as a continuous variable (months), and the ALSFRS-R bulbar subscore ranged from 0 to 12, with lower scores indicating greater bulbar impairment.*
